# MACHINE LEARNING ANALYSIS OF MOUSE FRAILTY FOR PREDICTION OF BIOLOGICAL AGE AND LIFE EXPECTANCY

**DOI:** 10.1093/geroni/igz038.3299

**Published:** 2019-11-08

**Authors:** Alice Kane, Michael B Schultz, Sarah Mitchell, Michael MacArthur, James Mitchell, Susan E Howlett, Michael S Bonkowski, David Sinclair

**Affiliations:** 1 Paul F. Glenn Center for Biology of Aging Research at Harvard Medical School, Boston, Massachusetts, United States; 2 Harvard T.H. Chan School of Public Health, Boston, Massachusetts, United States; 3 Dalhousie University, Halifax, Canada; 4 Department of Dermatology, The Feinberg School of Medicine, Northwestern University, Evanston, Illinois, United States

## Abstract

In mammals, the lack of accurate biomarkers for biological age is a current limitation to identifying novel aging interventions. Molecular biomarkers including DNA methylation hold promise but are invasive and currently expensive. The Frailty Index (FI) quantifies the accumulation of health-related deficits and is fast, cheap, and non-invasive. Studies have demonstrated that FI correlates with age and mortality risk in mice and humans. However, the FI has not been modelled to directly predict biological age or life expectancy. We tracked aging male C57BL/6 mice until their natural deaths, scoring them longitudinally with the FI. We find that FI score correlates with and is predictive of age and that some but not all parameters of the FI are individually well-correlated with age. To better predict chronological age, we performed an elastic net regression on the FI termed FRIGHT (Frailty Inferred Geriatric Health Timeline) Age. FRIGHT Age is a strong predictor of age (r2=0.73, median error=47.5 days), but is not superior to chronological age at predicting life expectancy. To better predict mortality, we built a random forest model termed the AFRAID (Analysis of Frailty and Death) score, which predicted survival at multiple ages (r2=0.375, median error = 46.4 days). The FRIGHT and AFRAID models were responsive to chronic treatment with enalapril (30mg/kg/day), an angiotensin converting enzyme inhibitor that extends healthspan, and methionine restriction, a dietary intervention that extends healthspan and lifespan. Our findings underscore the value of assessing non-invasive biomarkers for aging research and may help speed the identification of aging interventions.

